# Remarks on M. Lemaire's Paper Concerning a Peculiar Structure of the Teeth

**Published:** 1817-06

**Authors:** John Nicholles


THE LONDON

### Medical and Physical Journal.

6 OF VOL. XXXVII.] JUNE, 1817. [NO. 220.

" For many fortunate discoveries in medicine, and for the detection of nume-
" rous errors, the world is indebted to the rapid circulation of Monthly
" Journals; and there never existed any work to which the Faculty in
" EUROPE and AMERICA were under deeper obligations than to the
" Medical and Physical Journal of London, now forming a long, but an
" invaluable, series."—RUSH.

*For the London Medical and Physical Journal.*

### *Remarks on M. Lemaire's Paper concerning a peculiar Structure of the Teeth;* by JOHN NICHOLLES, Esq.

YOUR note to the remarks "On some Peculiarities of the Teeth, by M. Lemaire, Dentist, of Paris," in the Number of the Medical and Physical Journal for February last, is chargeable with whatever trouble this communication may occasion you.

I should have considered that gentleman's laconic paper undeserving of attention, especially when the character of your readers is considered, were it not for his illiberal abuse of a lately departed ornament of society—I mean Mr. Joseph Fox.

Upon examining the mouth of the young lady, the subject of M. Lemaire's "peculiarity of structure," he informs us that "he found the temporary cuspidatus had been extracted to make room for the secondary incisores, as is now the common practice." Is this a suggestion that the common practice is erroneous? Does he not know that the jaw rarely becomes early enough expanded to allow of the regular growth of the four incisores? consequently such "common practice" is supported by the best test—that of experience. Why the extraction of the temporary cuspidati should have been matter of surprise, when all the young lady's temporary teeth had been long extracted, cannot be well conceived, unless for the purpose of conveying oblique censure.

M. Lemaire proceeds—"The first small molaris had replaced the cuspidatus in such a manner, that the latter, meeting with obstacles, had not shewn itself between the first little molaris and the little *incisores* [incisor] until the young lady had reached her sixteenth year."

The former part of this remark is, again, incorrect. The first little molaris [bicuspis] had not replaced the temporary cuspidatus; it was the lateral incisor which occupied the situation in which it [the temporary cuspidatus] formerly stood.

As to the propriety of extracting either of the bicuspides or the cuspidatus, no one, without seeing the mouth, can determine; but, however plausible the "principle" may be, "of never deranging what appears to be well," it is only in cases where the first bicuspis is more than usually prominent, that the cuspidatus should be extracted, as it contributes so much to the beauty and expression of the countenance.

If I properly understand the remark—"with my instrument reversed, I made a demi-luxation," this cuspidatus was attempted to be extracted by the key-instrument, the use of which, in such cases, is at all times unnecessary, dangerous, and inconvenient.

But the great peculiarity of the case is, the "extreme surprise in finding *four* instead of *one cuspidatus.*" It is fortunate the plate accompanied this description, for otherwise it would certainly have been unintelligible. There were assuredly not *four cuspidati*—there was *one cuspidatus* and three small bony processes, or supernumerary teeth, incapable of ever resembling cuspidati. What seems to throw a doubt upon their being supernumerary teeth at all, is, the fact of M. Lemaire mistaking them for a piece of *tartar*, "being precisely of the same colour." Now we know extremely well, that, at this age, all new-formed teeth are particularly white: and we might, at least, expect them to be all of the same colour.

It appears that the parents of this young lady had neglected to consult any regular dentist until all the mischief that could happen was completed. Is it not, then, very probable that the temporary molaris, in this case, had very early become decayed, and, losing its crown, had left the three fangs in their own *alveoli;* which last, being gradually absorbed, would certainly leave these remnants very loose, and easily extracted? But, being unmolested, the young lady, perhaps, instead of complaining, taking care to keep them in, and submitting to masticate on the other side only, the bicuspis regularly appearing, and the cuspidatus pressing downwards, fixed them firmly, and ultimately concealed them from view. At this time, and in this condition, M. Lemaire very ingeniously removed them, with the cuspidatus, and, in *his* imagination, converted them into supernumerary teeth! Cases of this kind are seen every day; but dentists are seldom consulted upon them, unless they occasion pain by the the fangs producing pressure on the gums.—I think we are entitled to infer thus much, from the negligence with which M. Lemaire appears to view the productions of nature, as well as those of talent.

I send you, with this, a model* I took of a mouth, some short time since, in which you will perceive three perfectly-formed permanent cuspidati.

---

The note relative to the extraction of the Dens Sapientiæ, appended by M. Lemaire to his paper, is an unintended compliment to the memory of Mr. Fox, and proves how little M. Lemaire must have read, or, if he read, how little he understood, or, if he understood, how carefully he concealed the merits, of the "quarto volume, printed in a most expensive style, with 19 plates" (26 it has).

Mr. Fox particularly points out the absolute propriety of extracting the dens sapientiæ externally, and, for that purpose, has adopted and warmly recommended Mr. Spence's instrument. I shall give Mr. Fox's own words, as I cannot improve them.

"The extraction of the dentes sapientiæ of the under jaw is attended with more difficulty than that of any other of the teeth. The jaw-bone begins to rise on the outer side of the dentes sapientiæ, in order to form the coronoid process; and, in some persons, the rise of the bone is nearly as high as the tooth. Hence, there is not sufficient depth for the bolster of the instrument to be applied on the outside of the tooth, and there would always be a necessity for drawing the tooth inwardly, if we did not possess a safe method of extracting it outwardly. It was with this view that the improvement of Mr. Spence was introduced. A dens sapientiæ, which is much decayed on the outer side, could not be drawn inwardly, because the decayed state of the tooth would prevent proper hold for the instrument. Likewise, with a common key-instrument, it would not be possible to draw it outwardly, because the rise of the jaw-bone would render it impracticable. These difficulties are entirely obviated by advancing the claw beyond the bolster; a fulchrum is then conveniently obtained by applying the bolster upon the jaw at the outer side of the second molaris, and, the claw being fixed on the inner side of the tooth, it may be drawn outwardly with great safety. By operating in this manner, much difficulty is obviated, and the danger of breaking away large portions of the alveolar process is prevented."

---

* For a future Number, we are promised a drawing of the above, with Mr. Nicholles' remarks.

 Not-

Notwithstanding the excellence of these remarks as a general rule, and much as I have always esteemed the character and talents of the late author above quoted, I must remark, that my own experience has convinced me there are cases in which the dens sapientiæ can be extracted inwardly with much less pressure, and, consequently, less pain to the patient than outwardly; and also cases in which the tooth could not be extracted externally on account of its great inclination towards the tongue. In the case of M. Lemaire, the dens sapientiæ stood entirely alone; consequently, the plan of using Mr. Spence's instrument could not be adopted with success, as the means by which the fulchrum would have been obtained, viz. the second molaris, was gone. The tooth was decayed on the inside. The chance, therefore, of laying hold of it with the claw at that part was, in great measure, lost, even allowing that the rise of the jaw did not prevent the application of the bolster. But, says M. Lemaire, "the outside afforded ample space for attaching the forceps;" still he "experienced great difficulty in the operation of separating the splinter from the tooth," after what he calls a "demi-luxation" had taken place.

Attempts in general to extract any of the molares with the forceps are unavailing: the key-instrument, notwithstanding all the ingenious inventions and theories advanced, for the purpose of extracting teeth perpendicularly, is the most effectual, and, in the hands of a careful, intelligent, and experienced operator, the safest.

The fracture mentioned by M. Lemaire, the similar case in Mr. Fox's work, and one I myself saw some short time since, (the piece of which I have,) were not occasioned merely by the attempt to extract them externally, but by inattention to the size of the instrument. I mean particularly the claw, and the application of the bolster.

The plan recommended by Mr. Benjamin Bell, in his System of Surgery, "to press the bolster as low as possible," cannot be too severely reprobated, or too generally pointed out, as the work is usually read by students.*

In cases where it is necessary to extract the dens sapientiæ inwardly, care should be taken that the claw is not larger than is sufficient to embrace the tooth with ease. The bolster should be applied as nearly opposite the point of it as the eye can direct, and, whilst the pressure is sufficient

* Such are the unavoidable inconvenience of all *systems*, whether of surgery or medicine. Few men of sufficient knowledge have leisure for, or will attempt, such works.—Edit.

to move the tooth from its socket, an elevation should be performed by the hand, which would at all times remove the tooth with increased ease, and invariably prevent such dangerous occurrences.

*Fleet-street; May,* 1817.

---

*For the London Medical and Physical Journal.*

